# Investigation of *GSTP1* and *PTEN* gene polymorphisms and their association with susceptibility to colorectal cancer

**DOI:** 10.2478/raon-2025-0001

**Published:** 2025-01-04

**Authors:** Durr-e-Shahwar, Hina Zubair, Muhammad Kashif Raza, Zahid Khan, Lamjed Mansour, Aktar Ali, Muhammad Imran

**Affiliations:** 1 Biochemistry Section, Institute of Chemical Sciences, University of Peshawar, Peshawar, Pakistan; 2 Department of Chemistry, Shaheed Benazir Bhutto University Sheringal Dir upper, Sringeri, Pakistan; 3 Department of Zoology, College of Science, King Saud University, Riyadh, Saudi Arabia; 4 Biological Screening Core, Warren Family Center for Drug Discovery, University of Notre Dame, Notre Dame, United States

**Keywords:** colorectal cancer, GSTP1, PTEN, polymorphism, PCR-RFLP

## Abstract

**Background:**

This study investigates the association of single nucleotide polymorphism in glutathione S transferase P1 (rs1695 and rs1138272) and phosphatase and TENsin homolog (rs701848 and rs2735343) with the risk of colorectal cancer (CRC).

**Patients and methods:**

In this case-control study, 250 healthy controls and 200 CRC patients were enrolled. All subjects were divided into 3 groups: healthy control, patients, and overall (control + patients). Genotyping was performed using polymerase chain reaction-restriction fragment length polymorphism (PCR-RFLP). The demographic information, including age, gender, location, smoking status, cancer stage, and node involvement, were collected.

**Results:**

The allele frequencies of *PTEN* rs701848 in overall subjects were 0.78 for C and 0.22 for T. Similarly, in overall individuals, allele frequencies for *PTEN* rs2735343 were 0.65 and 0.35 for G and C alleles, respectively. The CC genotype or C allele of rs701848 and CG/GG genotype of rs2735343 were observed to be a risk factor for CRC. In overall individuals, a significant (p ≤ 0.05)) association was observed between rs701848 and rs2735343 polymorphisms CRC. Allele frequencies for *GSTP1* rs1695 were 0.68 and 0.32 for the A and G alleles, respectively. Allele frequencies for *GSTP1* rs1138272 were 0.68 and 0.32 for C and T alleles, respectively. However, a significant (p < 0.05) association was found in males for rs1695, while a non-significant difference was observed for the distribution of any genotypes or alleles at *GSTP1* (rs1138272).

**Conclusions:**

Both SNPs of *PTEN* rs701848 and rs2735343 polymorphisms were significantly associated with CRC. However, in *GSTP1*, rs1695 was significantly associated with CRC risk in males, and rs1138272 showed a non-significant association with colorectal cancer risk.

## Introduction

Colorectal cancer (CRC) is a major public health concern around the world, ranking among the top causes of cancer morbidity and mortality.^1^ CRC has the third-highest incidence and second-highest mortality rate of all cancers worldwide.^2^ Over 1918658 CRC cases and 900536 deaths were estimated in 2022.^3^ The incidence of CRC shows considerable variation among racially or ethnically defined populations in multiracial/ethnic countries.^4^ The geographical and temporal burden of this cancer provides insights into risk factor prevalence and progress in cancer control strategies.^5^ CRC causes include heterogeneous, controllable, and external factors related to lifestyle, such as diet and socioeconomic standing.^6^ Chromosomal instability (CIN) or microsatellite instability (MIN) are the two main causes of the development of CRC and involve activation and inactivation of various proto-oncogenes and tumor-suppressor genes, re-spectively.^7^ Several genes have been connected to the etiology of CRC including *GSTP1* (Glutathione S-Transferase Pi 1), *APC* (Adenomatous Polyposis Coli), and *PTEN* (Phosphatase and TENsin) *etc*.^8^

The *GSTP1* gene has six introns and seven exons and is positioned on chromosome 11q13. From aberrant crypt foci to advanced carcinomas, *GSTP1* is overexpressed in all stages of CRC.^9,10^ GSTP1 dimers catalyze the conjugation of glutathione’s sulfur atom to endogenous and exogenous electrophiles, such as xenobiotics, reactive oxygen species (ROS), anticancer agents, and carcinogens in the process of detoxification.^11,12^ Two important genetic polymorphisms in *GSTP1* include rs1695 (Ile-105Val) resulting from an AG transition at base 1578 (c.313A>G), and rs1138272 (Ala114Val), resulting from a CT transition at base 2293 (c.341C>T).^12^ These polymorphisms may predispose to CRC through deficient detoxification of carcinogens and also may have an impact on a patient’s response to chemotherapy.^13^

A tumor suppressor gene called *PTEN*, which codes for a protein that has both lipid and protein phosphatase functions, is found on chromosome 10q23.3.^14^ Blocking the oncogenic PI3K/Akt/mTOR pathway is the primary function of *PTEN*. Genetic alterations in *PTEN* leading to its inactivation, facilitate tumorigenesis, and are common in human cancers such as prostate cancer, breast cancer, glioblastoma, and CRC.^15^ Single nucleotide polymorphisms (SNPs) in *PTEN* can decrease its activity which may lead to downstream oncogene activation and tumorigenesis.^16^ The *PTEN* gene’s intron and non-coding region contain SNPs like rs2735343 (located in the promoter region of the gene, C > G change) and rs701848 (found in the 3′ untranslated region (3′-UTR) of the gene T>C change), which may affect splicing, cell cycle, and protein expression.^17^

These genetic polymorphisms can affect the enzymes by either modifying enzymatic activation, their interaction with partner proteins, or their detoxification potential, which can potentially influence the susceptibility and prognosis to CRC based on ethnic disparities and inter-individual differences. The association of these polymorphisms in the candidate genes with CRC risk in the Khyber Pakhtunkhwa population has not been established yet. This study was thus designed to investigate genetic/allelic polymorphism in *GSTP1* (rs1695, rs1138272) and *PTEN* (rs701848, rs2735343), their frequency, and their association with the development of CRC.

## Patients and methods

### Samples collection

In this study, 250 healthy controls and 200 CRC patients of various stages from I-IV under chemotherapy or radiation therapy treatments were enrolled from Khyber Pakhtunkhwa, Pakistan. The sample size was calculated World Health Organization (WHO) formula.^18^ Ethical approval for this study was obtained from the Ethical Committee Faculty of Life & Environmental Sciences, University of Peshawar, Pakistan. For the controls, healthy individuals with no sign of present or previous malignancy and no indication of CRC nor any family history of cancer were included who have no blood relation with the patients. Individuals who were unable to provide informed consent and patients who have developed CRC at the age > 60 years were excluded. Mixed ethnic backgrounds individuals and patients with comorbidities were also excluded. Blood samples (3 mL) were collected through a sterile syringe from both the patients and controls visiting the Institute of Radiation and Nuclear Medicine (IRNUM), Peshawar, Pakistan. They were stored at −20°C in sterile vacutainer tubes containing ethylenediaminetetraacetic acid (EDTA) till further analysis.

### DNA extraction and genotyping

Genomic DNA was extracted using a genomic DNA extraction kit (Gene JET Genomic DNA Purification kit, Thermoscientific, USA) and quantified using a spectrophotometer (752 PC, China). A 5–10 ng DNA sample was used for the genotyping of *GSTP1* (rs1695, rs1138272) and *PTEN*(rs701848, rs2735343) polymorphisms using the polymerase chain reaction-restriction fragment length polymorphism (PCR-RFLP) technique.^19, 20^ The PCR amplification was performed in a 25 mL reaction mixture, containing 100 ng genomic DNA, 0.2 mM dNTP, 0.2 mM of each primer, 2.5 U Taq DNA polymerase, and Taq buffer (Thermo Fischer Scientific USA). The primer sequences were designed using Primer 3 or Primer BLAST. The sequence of primers, PCR conditions, restriction enzymes, length of PCR, and digestion products for *GSTP1* and *PTEN* amplification have been described in Table 1. The PCR products were digested by respective restriction enzymes overnight at 37°C and then analyzed by electrophoresis on 2% agarose gel. The sequences of the PCR products were confirmed by Sanger sequencing. Sanger sequencing (capillary sequencing) of random samples was carried out using Applied Biosystems 3730xl DNA Analyzer (Thermo Fischer Scientific, USA). Bioedit sequence alignment editor (BioEdit version 7.7.1) was used for sequencing data analysis.

**TABLE 1.** Primer sequences and amplification conditions for *GSTP1* and *PTEN* polymorphisms

Gene	Primer sequence	PCR conditions	Amplicon length (bp)	Restriction enzyme	Length of digest products (bp)	Enzyme specificity
*GSTP1* (rs1695)	F:5′GGCTCTATGGGAAGGACCAGCAGG-3′ R:5′GCACCTCCATCCAGAAACTGGCG3′	30 cycles of 1 min at 94°C,1 min at 66°C and 2 min at 72°C.	445	*Alw*261	330+115+270	5’GTCTC(N)1↓3’ 3’CAGAG(N) 5↑5’
***GSTP1* (rs1138272)**	F:5′CAGCAGAGGCAGCGTGTGTGC-3′ R:5′CCCACAATGAAGGTCTTGCCTCC-3′	30 cycles of 1 min at 94°C, 1 min at 64°C and 2 min at 72°C.	565	*Aci*I	365+120+485+80	5’C↓CG↑C’3 **3’G↓GC↑G’5**
***PTEN* (rs701848)**	F:5’-GTGCTTTATTGATTTGCT-3’ R:5’AGTAGTTGTACTCCGCTT-3’	5 min at 94°C, 35 cycles of 30 s at 94°C, 30 s at 55°C, 30 s at 72°C, and 10 min extension at 72°C.	199	*HaeIII*	199+81+118	5’GG↑CC3’ 3’CC↑G G5’
***PTEN* (rs2735343)**	F:5’-CTCTTCCTGTTCTCCATCGTG-3’ R:5’-TTCTCCAGGATTTCGTCTGC-3’	5 min at 94°C, 35 cycles of 30 s at 94°C, 30 s at 63°C, 30 s at 72°C and 10 min at 72°C.	272	*HhaI*	272+72+200	5’G↑CG↑C3’ 3’C↑GC↑G’5

1 bp = base pair; F = forward; PCR = polymerase chain reaction; R = reverse

### Statistical analysis

The statistical package for social sciences version 20 (SPSSv.20) was used for analysis. Descriptive statistics were used to calculate proportions and percentages for each categorical variable used in univariate analysis. Adjusted odds ratios (OR) and 95% confidence interval (CI) for potential determinants of CRC were calculated by logistic regression analysis. The p ≤ 0.05 was considered to be statistically significant. Hardy Weinberg equilibrium was tested by chi-square t-test for observed genotype frequencies.

## Results

### Demographic variables of the studied population

Of the 450 individuals, 200 were CRC patients; 78 (39%) were female and 122 (61%) were males. The remaining 250 were controls; 81 (32%) female and 169 (68%) males. The inter-group differences related to age, gender, and food consumption patterns were non-significant (p > 0.05), while smoking status was a significant factor (Table 2). The tumor location among the CRC cases was non-significant (p > 0.05) as 98 (49%) patients were diagnosed with rectal carcinoma and 102 (51%) had colon carcinoma.

**TABLE 2.** Demographic information and risk factors in colorectal cancer (CRC) cases and control

Variable	Patients N = 200(%)	Control n = 250(%)	P value
*Age (Years)			
≥ 40	132 (66.0)	151 (60.4)	0.222
< 40	68 (34.0)	99 (39.6)	
Range	10-60	11-60	-
Median	46	32	-
*Gender			
Male	122 (61.1)	169(68)	0.273
Female	78 (39.0)	81(32)	
**Smoking status			
Never	165 (82.3)	22 (8.8)	< 0.010
Ever	35 (17.6)	228 (91.1)	
*Site of tumor			
Colon	102(51.00)		0.984
Rectum	98(49.00)		

* p > 0.05 patients vs control;

** p ≤ 0.05 patients vs controls

### Frequency of *GSTP1* (rs1695 and rs113828) polymorphism and associated risk of CRC

**FIGURE 1.**
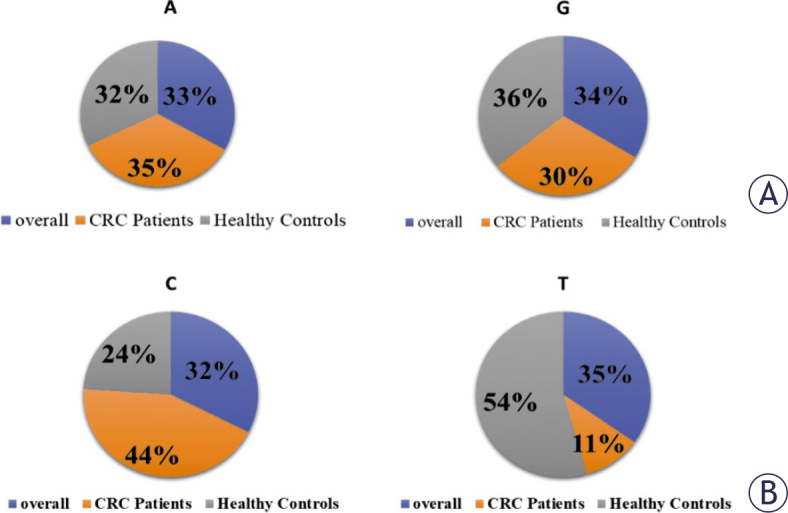
*GSTP1*Alleles frequency distribution of the rs1695 A/G **(A)** and rs1138272 C/T **(B)**. CRC = colorectal cancer

**FIGURE 2.**
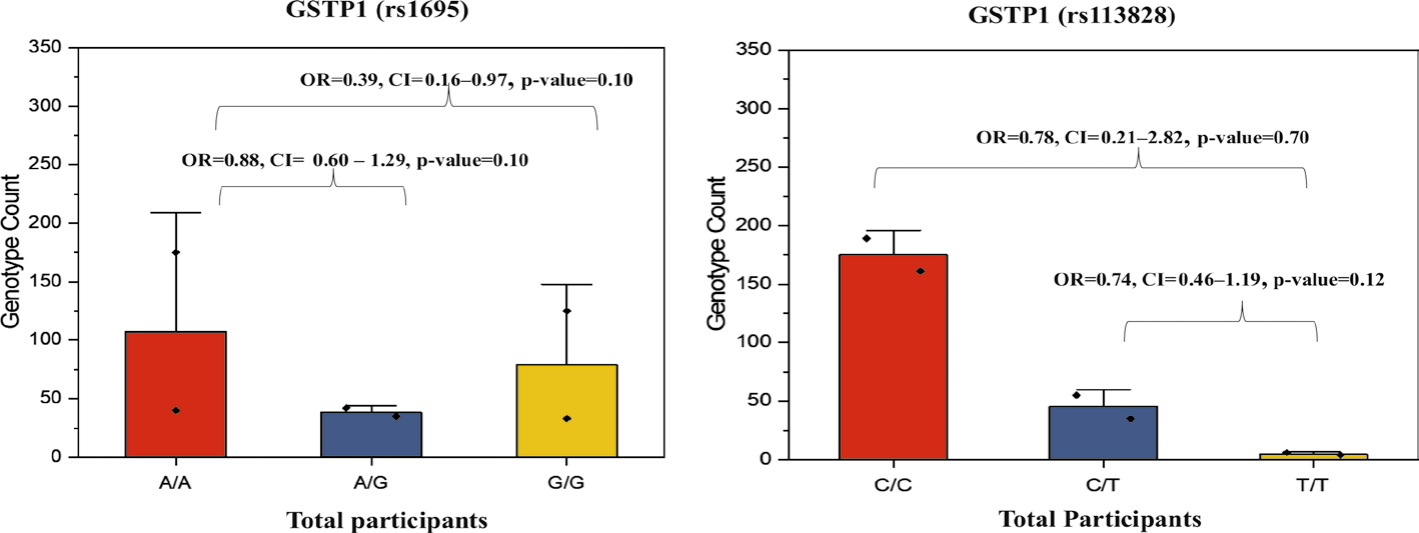
*GSTP1* genotypic count of the overall participants for rs1695 and rs1138272. The p-values and odds ratio (OR) displayed in the figure correspond to pairwise comparisons of genotypes in between the two groups. Lines typically represent trends or connections between data points and square dots mark data points or average. Genotype count means number of individuals with a specific genetic variation. CI = confidence interval

The risk association of rs1695 polymorphism and CRC is shown in Table 3. Representative images of genotyping are shown in Supplementary Figure 1 and random sample sequencing analysis is in Supplementary Figure 2. Among the 450 individuals, the A allele carriers of rs1695 were more prevalent compared to the G allele carriers. Allele and genotype frequency distribution for *GSTP1* in the population are shown in Figure 1 and Figure 2 respectively. In overall individuals, allele frequencies for *GSTP1* rs1695 were 0.68 and 0.32 for the A and G alleles, respectively. The genotypes (A/G+G/G) were not associated with the risk of CRC in overall subjects (OR = 0.81, CI = 0.56 to 1.19, P = 0.28, as well as in females (OR = 1.09, CI = 0.58 to 2.04, P = 0.79,) while it was associated in males (OR = 0.81, CI = 0.50 to 1.31, P < 0.01,). The relative risk (RR) for male was 2.2 times higher than for female participants.

**TABLE 3.** Frequency of *GSTP1* (rs1695) polymorphism and its association with colorectal cancer (CRC) risk

Models/Genotype	CRC Patients + Healthy Controls n (%)	CRC Patients n (%)	Healthy Controls n (%)	OR	P Value	95% CI	RR
**Overall Subjects**							
**Codominant Model**							
**A/A**	192 (43)	91 (46)	101 (40)		Referent		^_^
**A/G**	231 (51)	102 (51)	129 (52)	0.88	0.10	0.60–1.29	1.2
**G/G**	27 (6)	07 (4)	20 (8)	0.39	0.10	0.16–0.97	0.2
**Dominant Model (A/G+G/G)**	258 (57)	109 (54)	149 (60)	0.81	0.28	0.56–1.19	1.4
**Recessive Model (A/A+A/G)**	423	193	230	2.39	0.05*	0.99–5.79	-
**Over dominant Model (A/G)**	231 (51)	102 (51)	129 (52)	1.02	0.89	0.70–1.48	-
**Male**							
**A/A**	123 (42)	58 (48)	65 (38)		Referent		^_^
**A/G**	150 (52)	63 (52)	87 (51)	0.81	< 0.01*	0.50–1.31	1.3
**G/G**	18 (6)	01 (0.1)	17 (1)	0.07	< 0.01*	0.01–0.51	0.2
**Dominant Model (A/G+G/G)**	168 (58)	64 (52)	104 (52)	0.69	0.12	0.43–1.11	1.5
**Female**							
**A/A**	69 (43)	33 (42)	36 (44)		Referent		^_^
**A/G**	81 (51)	39 (50)	42 (52)	1.01	0.55	0.53–1.93	1.1
**G/G**	9 (6)	06 (8)	03 (4)	2.18	0.55	0.50–9.43	0.1
**Dominant Model (A/G+G/G)**	90 (57)	45 (58)	45 (56)	1.09	0.79	0.58–2.04	1.2
**HWE (Genotype Frequencies)**						
**A^2^**	0.46	0.50	0.436	-	-	-	
**2AG**	0.43	0.41	0.449	-	-	-	
**G^2^**	0.10	0.08	0.116	-	-	-	
**χ^2^total = 1**							

* Statistically significant associations (p ≤ 0.05), Logistic regression model adjusted by age, gender and smoking;

1 CI = confidence interval; CRC = colorectal cancer; OR = odd ratio; RR = relative risk

The risk association of *GSTP1* rs1138272 and CRC is shown in Table 4. Allele and genotype frequencies for *GSTP1* rs1138272 in the population are shown in Figure 1, 2 respectively. In overall subjects, the T allele (35%) was more prevalent than C allele (32%). In overall subjects the presence of the genotype C/T+T/T (OR = 0.75, CI = (0.47 to 1.18), P = 0.21) was not related to the risk of CRC and the relationship is not significant. Relative Risk for male and female is equal.

**TABLE 4.** Frequency of *GSTP1*(rs1138272) polymorphism and its association with colorectal cancer (CRC) risk

Models/Genotype	CRC Patients +Healthy Controls n (%)	CRC Patients n (%)	Healthy Controls n (%)	OR	P Value	95% CI	RR
**Overall Subjects**						
**Codominant Model**						
**C/C**	350 (78)	161(80)	189(76)		Referent		^_^
**C/T**	90 (20)	35(18)	55(22)	0.74	0.12	0.46-1.19	0.2
**T/T**	10 (2)	4(2)	6 (2)	0.78	0.70	0.21-2.82	0.03
**Dominant Model C/T+T/T**	100 (22)	39 (19)	61 (24)	0.75	0.21	0.47-1.18	0.3
**Recessive Model (C/C+C/T)**	440	196	244	1.20	0.77	0.33-4.32	-
**Over dominant Model**						
**(C/T)**	90 (20)	35(18)	55(22)	0.75	0.23	0.46-1.20	-
**Male**							
**C/C**	229(79)	98(80)	131(78)		Referent		-
**C/T**	56(19)	20(17)	36(21)	0.74	0.33	0.40-1.36	0.2
**T/T**	6(2)	4(3)	02(1)	2.67	0.26	0.47-14.89	0.02
**Dominant Model C/T+T/T**	62(21)	24(20)	38(22)	0.84	0.56	0.47-1.49	0.2
**Female**							
**C/C**	121(76)	61(78)	60(74)		Referent		^_^
**C/T**	34(21)	15(19)	19(24)	0.77	0.51	0.36-1.66	0.3
**T/T**	4(3)	2(3)	02(2)	0.98	0.98	0.13-7.21	0.04
**Dominant Model C/T+T/T**	38(24)	17(22)	21(26)	0.88	0.74	0.43-1.82	0.3
**HWE (Genotype Frequencies)**						
**C^2^**	0.462	0.81	0.25	-	-	-	-
**2CT**	0.435	0.18	0.5	-	-	-	-
**T^2^**	0.102	0.01	0.25	-	-	-	-
**χ^2^total = 1**							

* Statistically significant associations (p ≤ 0.05), Logistic regression model adjusted by age, gender and smoking.

1 CI = confidence interval; CRC = colorectal cancer; OR = odd ratio; RR = relative risk

**TABLE 5.** Frequency of *PTEN* (rs701848) polymorphism and its association with colorectal cancer (CRC) risk

Models/Genotype	CRC Patients +Healthy Controls n (%)	CRC Patients n (%)	Healthy Controls n (%)	OR	P Value	95% CI	RR
**Overall Subjects**							
**Codominant Model**							
**T/T**	293 (65)	136 (68)	157 (63)		Referent		^_^
**T/C**	113 (25)	30 (15)	83 (33)	0.41	0.03*	0.25–0.67	0.5
**C/C**	44 (10)	34 (17)	10 (4)	3.92	0.03*	1.86– 8.23	0.06
**Dominant**							
**T/C+C/C**	157 (35)	64 (32)	93 (37)	0.79	0.25	0.53–1.17	0.5
**Recessive Model (T/T+T/C)**	406	166	240	0.20	< 0.01*	0.09–0.42	-
**Over dominant Model**							
**(T/C)**	113 (25)	30 (15)	83 (33)	0.35	< 0.01*	0.22–0.56	-
**Male**							
**T/T**	197 (68)	86 (70)	111 (66)		Referent		
**T/C**	71 (24)	17 (14)	54 (32)	0.40	0.04*	0.21–0.75	^_^
**C/C**	23 (8)	19 (16)	04 (2)	8.02	0.01*	2.29-28.01	0.04
**Dominant**							
**T/C+C/C**	94 (32)	36 (30)	58 (34)	0.81	0.42	0.49–1.35	0.5
**Female**							
**T/T**	96 (60)	50 (64)	46 (57)		Referent		^_^
**T/C**	42 (27)	13 (17)	29 (36)	0.41	0.02*	0.19–0.89	0.6
**C/C**	21 (13)	15 (19)	06 (7)	2.05	0.14	0.77-5.48	0.1
**Dominant T/C+C/C**	63 (40)	28 (36)	35 (43)	0.72	0.32	0.38–1.36	0.7
**HWE (Genotype Frequencies)**							
**T^2^**	0.04	0.04	0.03	-	-	-	
**2TC**	0.34	0.32	0.29	-	-	-	
**C^2^**	0.60	0.64	0.67	-	-	-	
**χ^2^total = 1**							

* Statistically significant associations (p < 0.05), Logistic regression model adjusted by age, gender, and smoking.

1 CI = confidence interval; CRC = colorectal cancer; OR = odd ratio; RR = relative risk

### Frequency of *PTEN* (rs701848) polymorphism and associated risk of CRC

The allele frequencies of *PTEN* rs701848 in overall subjects were 0.78 for C and 0.22 for T. Representative images of genotyping are shown in Supplementary Figure 2 and random samples sequencing in Supplementary Figure 4. Among overall 450 subjects, the C allele was more prevalent compared to T allele carriers. The presence of C/C genotype was significantly associated with a higher risk of CRC in overall subjects (OR = 3.9, CI = 1.86 to 8.23, P = 0.03 in males (OR = 8.02, P = 0.001, CI = 2.29 to 28.01) as well as in females (OR = 2.05, P = 0.14, CI = 0.77 to 5.48). The RR for females was 0.8 times higher than males.

**FIGURE 4.**
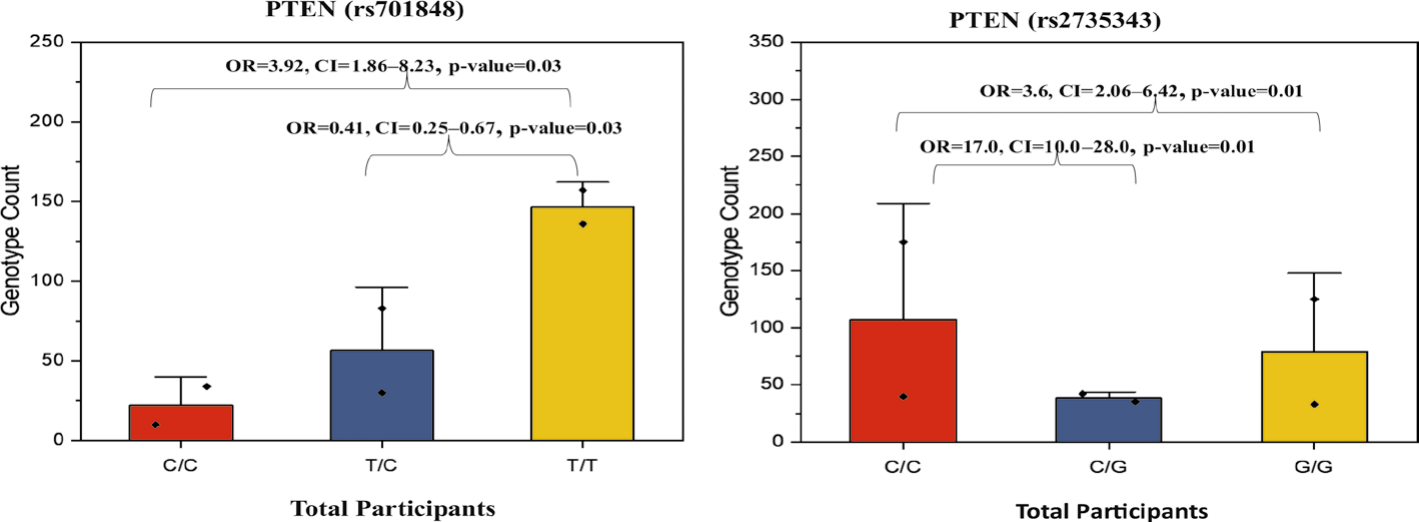
*PTEN* genotypic count of the overall participants for rs701848 and rs2735343. The p-values and odds ratio (OR) displayed in the figure correspond to pairwise comparisons of genotypes between the two groups in each of the bar graphs. Lines typically represent trends or connections between data points and square dots mark data points or average. Genotype count means number of individuals with a specific genetic variation. CI = confidence interval

### Frequency of *PTEN* (rs2735343) polymorphism and associated risk of CRC

**FIGURE 3.**
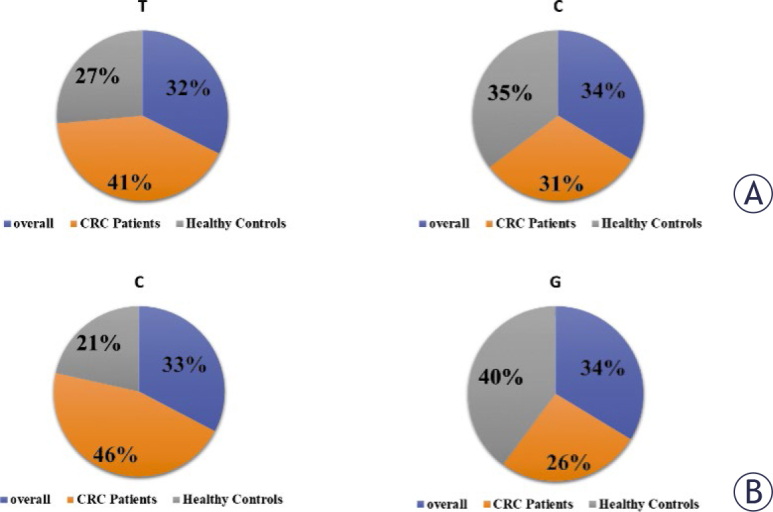
*PTEN* Alleles frequency distribution of the rs701848 T/C **(A)** and rs2735343 C/G **(B)**. CRC = colorectal cancer

The risk association of CG rs2735343 polymorphism and CRC is shown in Table 6. Similarly, in overall individuals, allele frequencies for *PTEN* rs2735343 were 0.65 and 0.35 for G and C alleles, respectively. In overall subjects, the G allele (C/G+G/G) was more prevalent (52%) than the C/C genotype (48%). The combined heterozygous C/G+G/G variant was observed to be 30% prevalent in healthy individuals and 80% in CRC participants. The allele frequencies and genotype count for rs2735343 are presented in Figure 3,4. The presence of genotypes (C/G, GG & C/G+G/G) was positively correlated with a higher risk of CRC in overall subjects, males and females (OR = 3.6-17.0). The RR for males is 2.8 times greater than females.

**TABLE 6.** Frequency of *PTEN* (rs2735343) polymorphism and its association with colorectal cancer (CRC) risk

Models/Genotype	CRC Patients +Healthy Controls n (%)	CRC Patients n (%)	Healthy Controls n (%)	OR	P Value	95% CI	RR
**Overall Subjects**							
**Codominant Model**							
**C/C**	215 (48)	40 (20)	175 (70)		Referent		^_^
**C/G**	77 (17)	35 (18)	42 (17)	3.6	< 0.01*	2.06–6.42	0.2
**G/G**	158 (35)	125 (62)	33 (13)	17.0	< 0.01*	10.0–28.0	0.1
**Dominant**							
**C/G+G/G**	235 (52)	160 (80)	75 (30)	9.5	< 0.01*	6.10–14.7	0.4
**Recessive Model (C/C+C/G)**	292	75	217	0.09	< 0.01*	0.05–0.14	-
**Over dominant Model**							
**(C/G)**	77 (17)	35 (18)	42 (17)	1.09	0.71	0.67–1.79	-
**Male**							
**C/C**	139 (48)	24 (20)	115 (68)		Referent		^_^
**C/G**	47 (16)	16 (13)	31 (18)	2.47	< 0.01*	1.17–5.22	0.2
**G/G**	105 (36)	82 (67)	23 (14)	17.08	< 0.01*	9.02–32.3	0.2
**Dominant**							
**C/G+G/G**	152 (52)	98 (80)	54 (32)	8.70	< 0.01*	5.01–15.0	0.4
**Female**							
**C/C**	76 (48)	16 (21)	60 (74)		Referent		^_^
**C/G**	30 (19)	19 (24)	11 (14)	6.48	< 0.01*	2.57–16.3	0.1
**G/G**	53 (33)	43 (55)	10 (12)	16.12	< 0.01*	6.68–38.9	0.1
**Dominant**							
**C/G+G/G**	83 (52)	62(79)	21 (26)	11.07	< 0.01*	5.28–23.3	0.3
**HWE (Genotype Frequencies)**							
**C^2^**	0.12	0.24	0.05	-	-	-	
**2CG**	0.45	0.5	0.35	-	-	-	
**G^2^**	0.42	0.26	0.59	-	-	-	
**χ^2^total = 1**							

* Statistically significant associations (p < 0.05), Logistic regression model adjusted by age, gender and smoking.

1 CI = confidence interval; CRC = colorectal cancer; OR = od Ratio; RR = relative risk

### Association of *GSTP1* and *PTEN* polymorphism with colon and rectum cancer cases

The study analyzed CRC patients based on tumor location to assess the association of the *GSTP1* and *PTEN* polymorphism and the link between the these polymorphisms and CRC was evaluated by sub-grouping the patients into those with colon and rectum cancers (Table 7). Of the 200 CRC patients, 102 (51%) had colon cancer and 98 (49%) had rectal cancer. Heterozygous genotypes were significantly linked to increased risks of both colon and rectal cancer (P < 0.05) for *GSTP1*(rs1695, rs1138272) and *PTEN* (rs701848).

**TABLE 7.** Association of *GSTP1* and *PTEN* polymorphism with colon and rectum cancer cases

Gene/rs	Genotype	Colon n = 102 (%)	Rectum n = 98 (49%)	P Value
***GSTP1* rs1695**	AA	62 (61.11)	26 (27.00)	Referent
	AG	34 (33.33)	72 (73.33)	< 0.01*
	GG	06 (5.65)	-	0.25
***GSTP1* rs1138272**	CC	91 (89)	69 (69.5)	Referent
	CT	13 (13.0)	17 (17.3)	< 0.01*
	TT	17 (17.0)	17 (17.3)	0.27
***PTEN* rs701848**	TT	72 (70.0)	64 (65.4)	Referent
	TC	13 (13.0)	17 (17.3)	< 0.01*
	CC	17 (17.0)	17 (17.3)	0.02
***PTEN* rs2735343**	CC	20 (19.6)	20 (20.4)	Referent
	CG	19 (18.6)	16 (16.3)	0.71
	GG	63(61.8)	62 (63.3)	0.96

* Statistically significant associations (p < 0.05)

## Discussion

CRC is a major global health issue influenced by various genetic factors. *GSTP1*, part of phase II detoxification, conjugates glutathione to detoxify and remove harmful substances, promoting detoxification.^21^ Polymorphisms in these genes alter biological pathways and protein expression, contributing to tumor development.^22^ *GSTP1* genotypes differin their ability to detoxify toxic species, with enzyme activity being significantly lower in individuals with Val instead of isoleucine at position 105 (rs1695).^23^ Research links *GSTP1* Ile105Val (rs1695, A>G) and *GSTP1* Ala114Val (rs1138272, C>T) mutations to various cancers, including breast, oral, and squamous cell carcinoma (SCC).^24^ *GSTP1* Ile105Val (rs1695, A > G) is a missense mutation reducing enzyme activity. Santric found a significant association between *GSTP1* Ile105Val polymorphism and toxicity.^25^ showed that the Kudhair *GSTP1* Ile105Val substitution increases lung cancer risk in Arab population. Watson *et al*. demonstrated that individuals with two *GSTP1* valine alleles had lower catalytic activity than those with two isoleucine alleles, with heterozygotes showing intermediate activity. Evidence on GST polymorphisms’ role in CRC susceptibility is mixed.^26^ *GSTP1*, highly expressed in the colon and involved in heterocyclic amine deactivation, is a candidate susceptibility gene. *GSTP1* SNPs, especially Ile105Val, are strongly associated with increased CRC risk and poorer prognosis. However, the association with rectal cancer is less robust than with colon cancer.^27^

The *GSTP1* gene variants (rs1695, rs1138272) are unlikely to significantly increase CRC risk, although a minor effect cannot be excluded, aligning with Terrazzino^27^ and Osti’s findings.^28^ The *GSTP1* 105Val allele frequency in CRC patients was similar to previous reports in healthy Caucasians and African-Americans.^29^ The frequency of both *GSTP1* polymorphisms was comparable to Australian, English, and American Caucasians (34%, 33%, and 33% Val-105; 7%, 8%, and 9% Val-114, respectively).^17^ Khabaz in Saudi Arabia, and studies in Bulgaria and Kashmir populations also found no association between these genotypes and CRC risk.^30^ However, Gorukmez^31^ noted the *GSTP1* Ile/Ile genotype was more frequent in controls than patients, while Vlaykova^32^ reported a non-significant protective role for the Val allele. A mild association of CRC with heterozygous and homozygous genotypes was observed compared to the wild type of GSTP1.^30^ Previous studies examining the Ile-1053Val and Ala-1143Val *GSTP1* polymorphisms in CRC reported no association, consistent with our findings.^33,34^

Phosphatase and TENsin homolog (*PTEN*) is also mutated in multiple advanced cancers and a tumor suppressor gene.^35^ *PTEN* is generally cytosolic and regulates phosphatidylinositol 3,4,5-trisphosphate (PIP3) levels; a small fraction of *PTEN* is recruited to the plasma membrane. *PTEN* reduces PIP3 levels, decreasing the mTOR/AKT signaling pathway critical for cancer cell growth, survival, and progression. Many SNPs and deletion polymorphisms in *PTEN* have been reported in human cancers.^36^ Both rs701848 and rs2735343 SNPs are located in the intron and non-coding region of the *PTEN* gene and increase cancer risk by probably influencing splicing, protein expression, and cell cycle. The rs701848 polymorphism influences cancer susceptibility by altering *PTEN* expression and reducing *PTEN* mRNA stability. These functional genetic polymorphisms of *PTEN* are known to participate in tumorigenesis.^30^ Jang *et al*^37^. and Xu *et al*.^38^ showed that the C allele of rs701848 was more susceptible than the T allele in developing esophageal squamous cell cancer (ESCC). The rs701848 is associated with an increased risk of breast cancer, renal cell cancer, CRC, and ESCC.^31^ GG genotype of rs2735343 is associated with an elevated risk of ESCC while there is no association between rs2735343 (G/C) and the risk of endometrial cancer. Moreover, Asian subjects carrying the TC/CC genotype or C allele of rs701848 were associated with an increased risk of esophageal squamous cell cancer.^16^ Studies have suggested a significant association between rs701848 and colon cancer risk, especially in populations with a family history of CRC. rs1903858 (G/A) and its specific association with colon, rectal, or CRC is still being researched, it has been implicated in cancer susceptibility in various populations.^39^ Located in the promoter region of *PTEN*, some studies suggest rs2735343 plays a role in both colon and rectal cancers through its impact on *PTEN* expression.^40^

Our analyses demonstrated that CRC risk was associated with rs701848 in the C/C genotype and with rs2735343 in the GG and C/G genotypes and shown that these genotypes increased the risk of CRC in the Pashtun population which supports previous findings by Jang *et al*.^37^ The distribution of genotypes or alleles in cases at both genetic sites of *PTEN* was statistically different from those in controls. This study is limited by a small Pashtun sample, lack of population comparisons, and no meta-analyses. Future research should replicate these findings in larger, multi-ethnic cohorts to assess genetic links to CRC. Investigations should focus on the effects of rs701848 and rs2735343 polymorphisms on PTEN expression and function to aid in developing targeted therapies.

## Conclusions

The significant association of *PTEN* rs701848 and rs2735343 polymorphisms CRC suggests their potential role as genetic risk factors in the studied population. The gender-specific association of *GSTP1* rs1695 with CRC in males warrants further investigation to elucidate the underlying mechanisms. These findings contribute to the understanding of genetic susceptibility to CRC and highlight the importance of personalized approaches in cancer prevention and treatment.

## Supplementary Material

Supplementary Material Details
